# Degradation of 2,4-Dichlorophenoxyacetic Acid (2,4-D) by Novel Photocatalytic Material of Tourmaline-Coated TiO_2_ Nanoparticles: Kinetic Study and Model

**DOI:** 10.3390/ma6041530

**Published:** 2013-04-15

**Authors:** Xuesen Bian, Jianqiu Chen, Rong Ji

**Affiliations:** 1 State Key Laboratory of Pollution Control and Resource Reuse, School of the Environment, Nanjing University, Nanjing 210023, China; E-Mail: woshjc@163.com; 2 Nanjing Institute of Environmental Sciences of the Ministry of Environmental Protection of PR China, Nanjing 210042, China; 3 Department of Environmental Science & Department of Analytical Chemistry, China Pharmaceutical University, Nanjing 210009, China

**Keywords:** novel photocatalytic material, tourmaline, TiO_2_, 2,4-D, model

## Abstract

The novel complex photocatalytic material was prepared by coating TiO_2_ nanoparticles on tourmaline using the sol-gel method, and used in the degradation of the herbicide 2,4-D. The results indicated that coating TiO_2_ with tourmaline enhanced the photocatalytic activity significantly. Based on the research of a simplified model for the average light intensity in the photoreactor, the influence of the concentration of photocatalyst, and the initial concentration of 2,4-D, a model for the degradation of 2,4-D by the tourmaline-coated TiO_2_ nanoparticles was established. Further tests showed that results calculated from this model were close to those obtained in the actual experiments.

## 1. Introduction

Photocatalysis has drawn a lot of attention in environmental application in recent years [1]. Removal of pollution from the environment using TiO_2_ as the photocatalyst is one of the research focuses due to TiO_2_’s innocuity, chemical stability, cheapness, commercial availability, reusability, as well as the vast reactivity towards wide variety of pollutants [2]. The reaction mechanism is usually explained as in [3], when absorbing photons holding energy equaling to or more than the band gap of TiO_2_, the electron will transit from its valence band to the conduction band, resulting in the separation of electrons and holes. The photogenerated holes can either oxidize the organic pollutants directly, or react with H_2_O molecules to form ^•^OH radicals, which show a strong oxidizability towards organic chemicals almost unselectively [4]. The photogenerated electrons may also react with H^+^ and oxygen dissolved in water to form H_2_O_2_, which can further be photo decomposed to ^•^OH radicals. Until now, the low quantum yield that arises by the easy recombination of photogenerated holes and electrons still limits the further broadening of practical utilization of photocatalytic techniques [5].

Tourmaline is a common natural mineral widely distributed in China. Since the discovery of its spontaneous electric field, this mineral has been utilized in areas like medical treatment, improvement in water quality, purification of air, *etc.* Investigations into its photochemical usage were carried out by some researchers, who attempted to employ it as an iron source of Fenton reagent [6,7]. The existence of the spontaneous electric field can help separate photogenerated holes and electrons. Thus, the mineral could be used to coat TiO_2_ nanoparticles to result in a better isolation of photogenerated holes and electrons, which could effectively enhance the quantum yield and thus the efficiency of photocatalytic reactions.

2,4-dichlorophenoxyacetic acid (2,4-D) is a type of phenoxy acid herbicide. 2,4-D and its salts as well as esters, are efficient, highly selective herbicides [8] and plant growth regulators [9]. It was initially registered in 1947 and, is still one of the most used herbicides in the world [10]. However, it may enter water bodies after usage in farmland [11], or for improper disposal [12], resulting in its broad residues in environment [13,14]. Exposure to this chemical has been proven to be harmful for the health of both humans and animals [15,16,17]. The degradation of 2,4-D in water is very slow, with a half-life of about 6 to over 170 days in different situations [18,19,20]. Therefore, the intentional removal of this chemical from water is considered necessary. Until now, many removal methods, e.g., adsorption [21,22], biodegradation [23], as well as photocatalytic degradation [24] have been investigated in previous studies. Among these methods, photocatalysis suits the demands of entire destruction of its chemical structure, the cheapness and good accessibility of treatment reagent, as well as the simplicity in operating conditions and techniques. This study is designed to investigate the improvement of TiO_2_ photocatalytic activity by tourmaline coating using 2,4-D as the target compound. For reference of the further utilization of this technology, a comprehensive model is initially developed to simulate the process based on research into effects of the common parameters of catalyst concentration, substrate concentration as well as the intensity of irradiation light.

## 2. Results and Discussion

### 2.1. Characterization of Tourmaline and Photocatalyst

**Figure 1:**
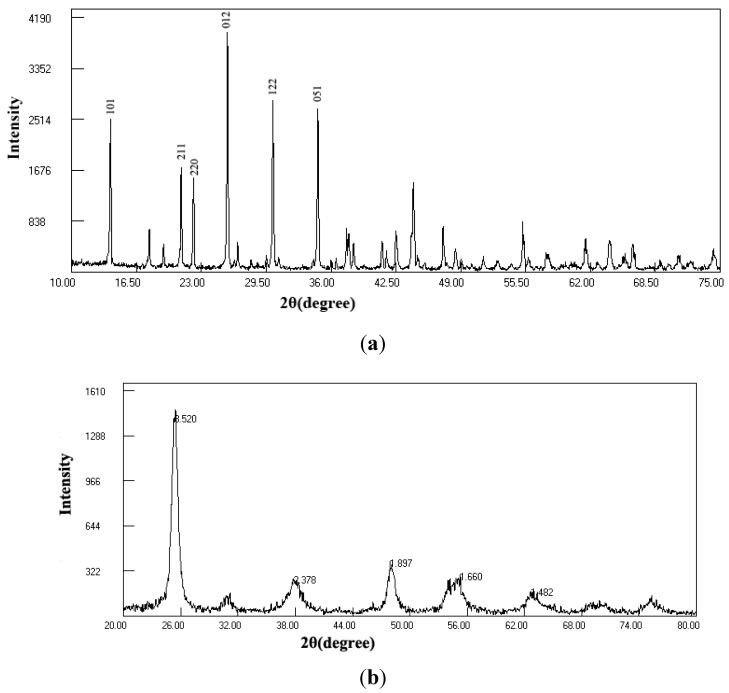
XRD spectra of (**a**) tourmaline; and (**b**) tourmaline-coated TiO_2_.

The XRD result of tourmaline used in this study is shown in Figure 1. The 2θ values of 13.88°, 20.96°, 22.20°, 30.14° and 34.66° are in accord with those from Meng *et al.* [25], demonstrating the characteristic peaks of Fe-tourmaline.

It is also shown in Figure 1 that tourmaline-coated TiO_2_ exists in the crystal type of anatase. The diameter of the TiO_2_ particles can be estimated according to the Scherrer formula: *D* = *Kλ*/*β*cos*θ*, in which *D* is the diameter (nm), *K* is the Scherrer constant and often valued as 0.9, *λ* is the wavelength of X-ray, valued 0.1541 nm in this study, *β* is the width at the half peak, and *θ* is the Bragg diffraction angle. The calculated *D* value for TiO_2_ in the complex photocatalyst is about 12 nm.

### 2.2. Photocatalytic Degradation

**Figure 2:**
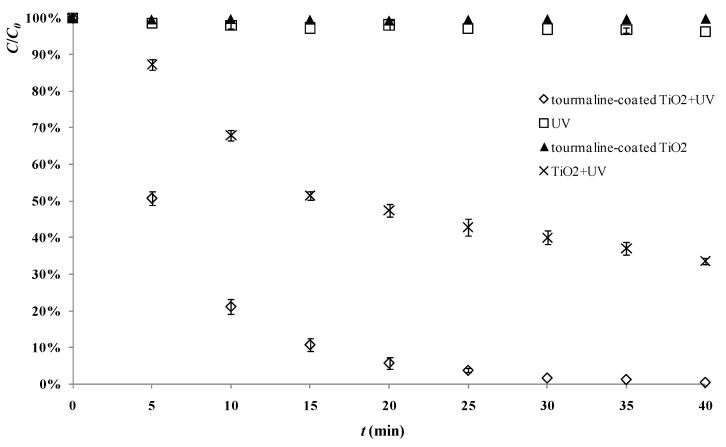
Photocatalytic degradation of 2,4-D, and control experiments without UV or catalyst (reaction conditions: air pumped in at 3 mL/s, *C_0_* = 20 mg/L).

Figure 2 shows degradation of 2,4-D in the presence of 500 mg/L tourmaline-TiO_2_ composite catalyst, with comparison to that with the same amount of pure TiO_2_. After 40 min, the photocatalytic degradation rate of 2,4-D by tourmaline-coated TiO_2_ was more than 90%, while the degradation rate by pure TiO_2_ was less than 70%. Figure 2 also shows that photodegradation of 2,4-D in the absence of photocatalyst, and adsorption of 2,4-D on tourmaline-TiO_2_ composite without UV irradiation was negligible. All these results indicate that tourmaline can effectively enhance the photocatalytic activity of TiO_2_ by coating.

### 2.3. Influence of the Initial Concentration of 2,4-D

Kinetic analyses indicate that the photocatalytic degradation rates of 2,4-D can usually be approximated using pseudo-first-order kinetics, according to the Langmuir–Hinshelwood model. In this case, the photocatalytic degradation reaction rate equation could be fitted by the pseudo-first order kinetics equation:

(1) −dC/dt = kC

(2) $$-\text{ln}C/C_{0}\;=\;kt\;+\;b$$

in which, *k* is the pseudo-first order reaction rate constant.

To examine the influence of the initial concentration of 2,4-D (*C_0_*), experiments were carried out with five different initial 2,4-D concentrations ranging from 20 mg/L to 100 mg/L, while all the other operating parameters were kept constant, as follows: tourmaline coated TiO_2_ concentration(*C_t_*) of 500 mg/L, air pumped in at 3 mL/s, and the UV light intensity at light source per unit length (*S_L_*) of 400 W/m.

**Figure 3:**
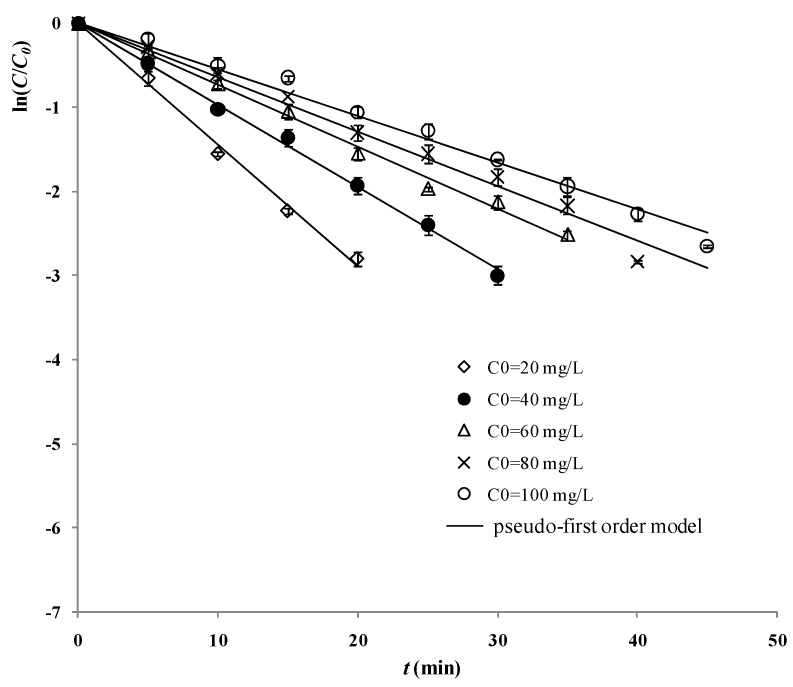
Logarithm values of 2,4-D residue ratios at different moments with various initial 2,4-D concentrations (reaction conditions: air pumped in at 3 mL/s, *C_t_* = 500 mg/L).

The logarithm values of 2,4-D residue ratios vs *t* at different initial 2,4-D concentrations are shown in Figure 3. The slope of a straight line for a plot of ln(*C/C_0_*) vs *t* is the opposite value of the pseudo-first order reaction rate constant (−*k*). Figure 3 shows that the lowering of initial concentration of 2,4-D led to the enhancement of the reaction rate constant *k*. This phenomenon might be explained by more absorption of photons by the increased 2,4-D molecules and/or the intermediates, which inhibited the activation of the photocatalyst.

**Figure 4:**
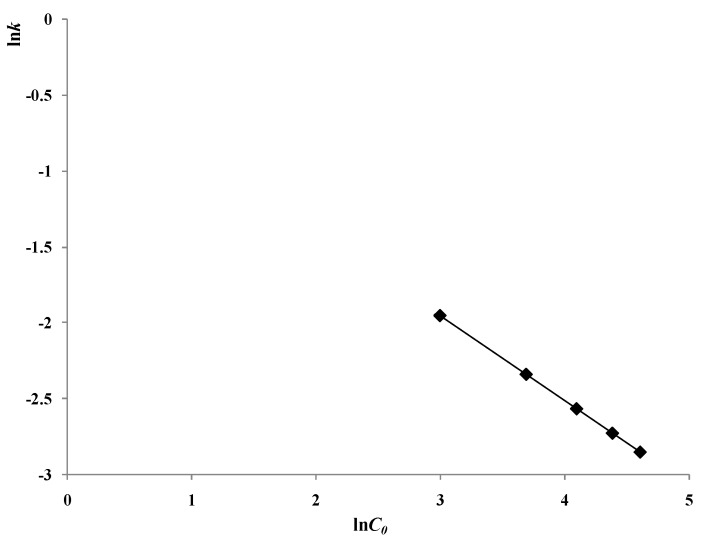
Varying trend of ln*k* to ln*C_0_* with various initial 2,4-D concentrations (reaction conditions: air pumped in at 3 mL/s, *C_t_* = 500 mg/L, *S_L_* = 400 W/m).

The varying trend of ln*k vs*. ln*C_0_* is shown in Figure 4, demonstrating a linear relationship between ln*k* and ln*C_0_* with a slop of −0.5594 (*R^2^* = 0.99).

So we can get the relationship between *k* and *C_0_*:

(3) $$K\;\propto \;{C_{0}}^{-0.5594}$$

There is an exponential relationship between the reaction rate constant *k* and the initial 2,4-D concentration.

### 2.4. The Light Intensity Distribution Model and the Influence of UV Light Intensity

The influence of UV light intensity was investigated by using various numbers of different 254 nm ultraviolet lamps, with the UV light intensities at *S_L_* of 200, 400, 600, 800, and 1000 W/m. All the other operating parameters were kept constant, as follows: tourmaline coated TiO_2_ concentration *C_t_* 500 mg/L, air pumped in at 3 mL/s, *C_0_* 20 mg/L.

Firstly, we need to establish a light intensity distribution model with a linear UV light source in a cylindrical reactor to investigate the influence of UV light intensity distribution of the circular cross-section area of the cylindrical reactor, and the light-shielding effect of the catalyst.

Two assumptions must be established before the development of the light geometric distribution model to make the process simple. Firstly, the lamp is a linear UV light source, and its diameter is negligible relative to the distance between the reactor and the light source; secondly, the attenuation of strong ultraviolet rays is mainly caused by absorption, reflection and scatter of turbid liquid in close range. Effects of other factors, such as air absorption and the reflector of the outer wall of the reactor, are negligible.

The average light intensity irradiating the cylindrical reactor can be calculated by the double integrals with the light source as the origin of the polar coordinate [7,26]:

(4) $$I_{ave}=\frac{1}{A}\int Ids=\frac{1}{A}\int \int I(r,\theta )rdrd\theta =\frac{1}{\pi R^{2}}\int_{r_{0}}^{r_{0}+2R}\int_{-\theta }^{\theta }I(r,\theta )rdrd\theta$$

The light intensity of a random point in the reactor *I*(*r, θ*) could be described as Equation (5):

(5) $$\frac{I(r,\theta )}{I(r_{1},\theta )}=\frac{r_{1}}{r}T$$

The light intensity at the wall of the photoreactor, *i.e.*, *I_0_*(*r_1_*,*θ*) is described as:

(6) $$I_{0}(r_{1},\theta )=\frac{S_{L}}{2\pi r_{1}}$$

*S_L_*, UV light intensity at light source per unit length W/m; *r_1_*, the distance between the reactor outer wall and the light source. In homogeneous liquid systems the light transmittance can be calculated by the Lambert-Beer law:

(7) T=exp(−A)

*A*, the absorbance of the systems:

(8) $$A=\varepsilon C_{t}L$$

The rigorous Lambert-Beer law only applies directly in homogeneous systems, while some deviation will take place when there is some nanometer catalyst powder [7,26]. However, the results of our experiments show that the Equation (9) could be approximately established when the concentration of nanometer catalyst powder *C_t_* is very low. The experiments were carried out using the UV-VIS Spectrophotometer, to analyze the absorbance *A* in the wavelength 254 nm lights with different *C_t_* and different *L* (the distance of the light travel through the reactor).

(9) $$T=\exp\left(-0.126C_{t}L\right)$$

**Figure 5:**
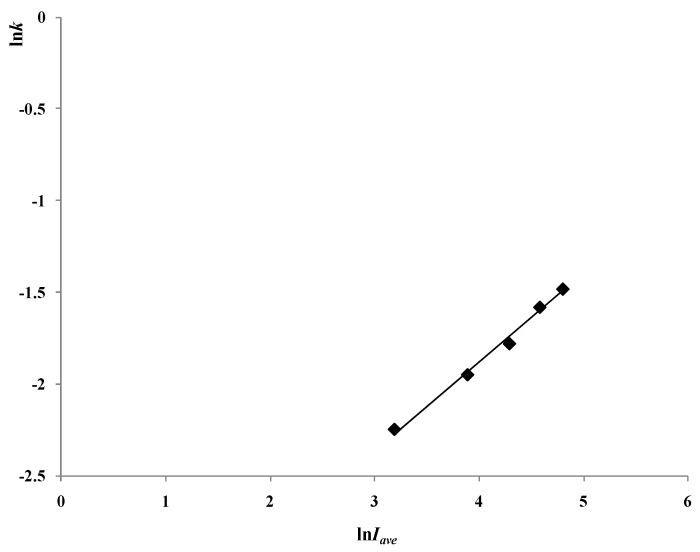
Varying trend of ln*k* to ln*I_ave_* with different light intensities (reaction conditions: air pumped in at 3 mL/s, *C_0_* = 20 mg/L, *C_t_* = 500 mg/L).

Using Simpson Integration, the average light intensity of the reactor *I_ave_* could be obtained with Equation (4). The varying trend of ln*k vs.* ln*I_ave_* is shown in Figure 5.

Figure 5 indicates that there is a linear relationship between ln*k* and ln*I_ave_*, with a slope of 0.4827 (*R^2^* = 0.99).

So the relationship between *k* and *I_ave_* could be given as Equation (10).

(10) $$K\;\propto \;{I_{ave}}^{0.4827}$$

### 2.5. The Influence of the Concentration of Photocatalyst

The influence of the photocatalyst concentration has two aspects: on one hand, the more photocatalyst is added, the more photons will be converted to chemical energy, and more ^•^OH and other strong oxidizing ingredients will be produced, which could accelerate the degradation of organic compounds. On the other hand, higher photocatalyst concentrations may lead to more reflection and scattering of photons in the system, which could depress the photodegradation of organic compounds.

**Figure 6:**
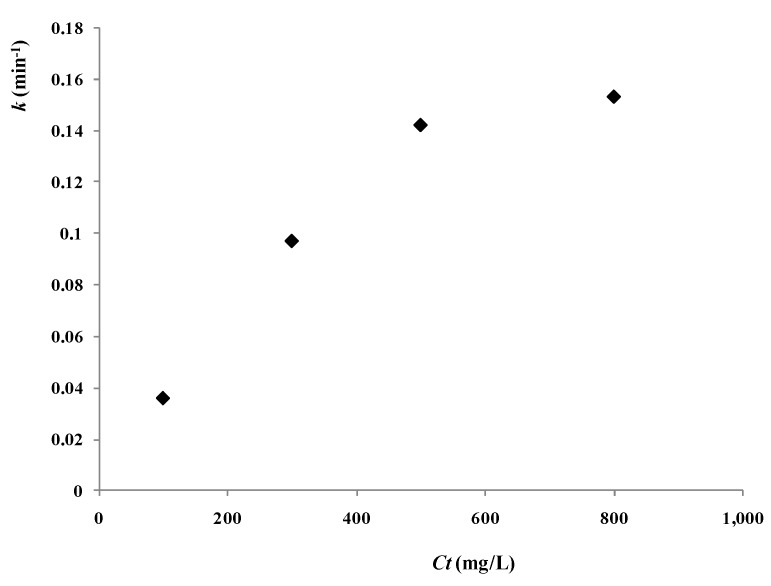
Varying trends of *k* to *C_t_* with different catalyst concentrations (reaction conditions: air pumped in at 3 mL/s, *C_0_* = 20 mg/L, *S_L_* = 400 W/m).

To examine the influence of catalyst concentration, the experiments were carried out with different doses of catalyst, and all the other operating parameters were kept constant, as follows: 2,4-D concentration *C_0_* 20 mg/L, air pumped in at 3 mL/s, and the UV light intensity at *S_L_* 400 W/m. The effect of catalyst concentration *C_t_* on the reaction rate constant *k* is shown in Figure 6. The photocatalytic reaction rate increased with the enhancement of the concentration of the catalyst; however, this trend of increase was gradually reduced because of the increase of reflection and scattering of photons by particles.

To examine the pure catalytic effect of the photocatalyst to the degradation process, the influence of the reflection and scattering of particles should be excluded. The reaction rate constant *k* in different catalyst concentrations was divided by *I_ave_*^0.4827^ to exclude the influence of the reflection and scattering of catalyst.

**Figure 7:**
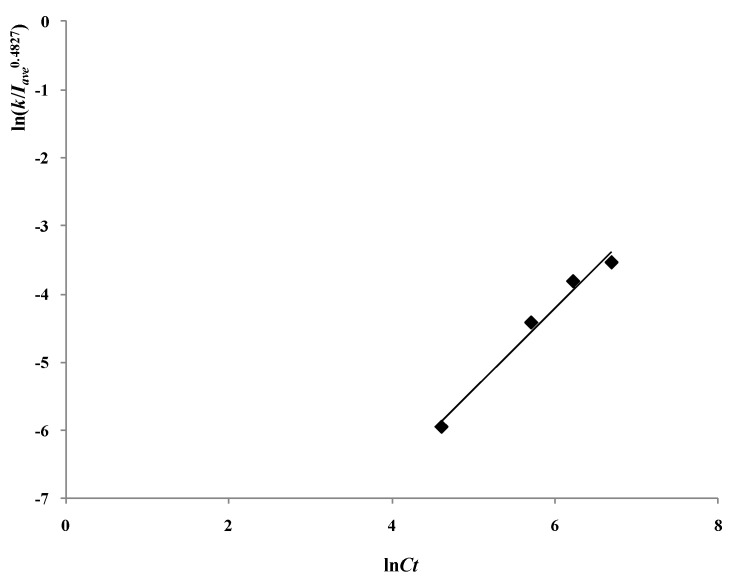
Varying trend of ln(*k/I_ave_*^0.4827^) to *lnC_t_* with different catalyst concentrations (reaction conditions: air pumped in at 3 mL/s, *C_0_* = 20 mg/L, *S_L_* = 400 W/m).

The varying trend of ln(*k/I_ave_*^0.4827^) vs ln*C_t_* at different catalyst concentrations is shown in Figure 7. There is a linear relationship between ln(*k/I_ave_*^0.4827^) and ln*C_t_* with the slope of 1.193 (*R^2^* = 0.98), so the relationship between the reaction rate constant *k* and average light intensity *I_ave_*, and the catalyst concentration *C_t_* is described as:

(11) $$K\;\propto \;{C_{t}}^{1.193}\cdot {I_{ave}}^{0.4827}$$

*I_ave_* is a function of the catalyst concentration *C_t_*, and it is reduced with the increase of *C_t_*.

### 2.6. Development of the Model and Tests in the Real Process

Based on the relationship shown in Equations (3), (10) and (11), the model can be developed for the degradation of 2,4-D by the tourmaline-coated TiO_2_ nanoparticles:

(12) $$k\;=\;K\;{C_{0}}^{-0.5594}\;{C_{t}}^{1.193}\cdot {I_{ave}}^{0.4827}$$

**Figure 8:**
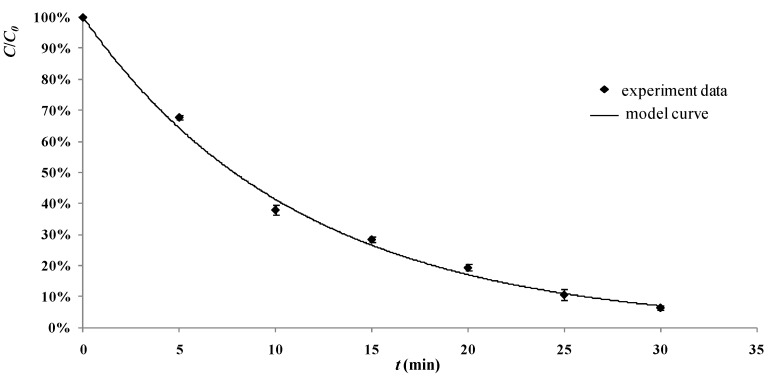
Values from the model developed in this study and from the actual experiments (reaction conditions: air pumped in at 3 mL/s, *C_0_* = 30 mg/L , *C_t_* = 400 mg/L, *S_L_* = 300 W/m)**.**

**Figure 9:**
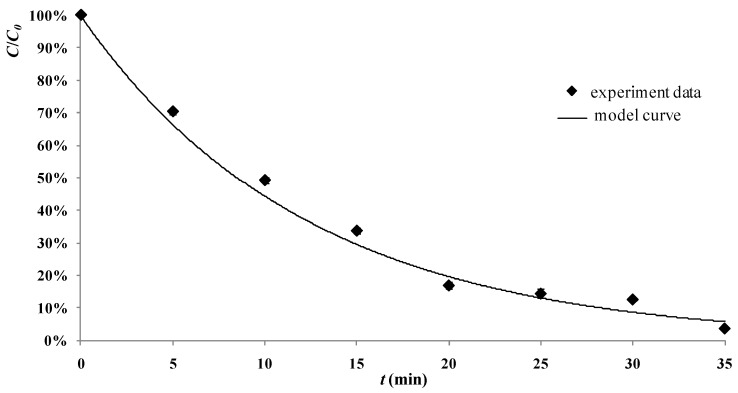
Values from the model developed in this study and from the actual experiments (reaction conditions: air pumped in at 3 mL/s, *C_0_* = 50 mg/L, *C_t_* = 600 mg/L, *S_L_* = 500 W/m)**.**

The degradation process of 2,4-D by the tourmaline-coated TiO_2_ nanoparticles could be estimated with Equation (12) using operating parameters *C_0_*, *C_t_* and *S_L_*. There were two experiments carried out with the operating parameters as follows: The first one was under condition of air pumped in at 3 mL/s, *C_0_* = 30 mg/L, *C_t_* = 400 mg/L, *S_L_* = 300 W/m; and the second one was under condition of air pumped in at 3 mL/s, *C_0_* = 50 mg/L, *C_t_* = 600 mg/L, *S_L_* = 500 W/m. The experimental data and the model calculation lines are shown in Figure 8 and Figure 9. It can be seen from the figures that results calculated from the model are close to their counterparts from the actual experiments.

## 3. Experimental Section

### 3.1. Materials and Instruments

2,4-D was purchased from Sigma–Aldrich Inc. (St. Louis, MO, USA) with a purity of 98%. Tourmaline was purchased from Chuanshi mineral processing factory, Hebei, China. Other chemicals were at least analytical grade, used without further purification. The UV light irradiation sources were several 100 W or 200 W UV lamps emitting monochromatic light at 254 nm purchased from Nanjing Huaqiang Electronic Co., LTD. The GC used in this study was an Agilent7890N series GC system with ECD detector. The column for separation was HP-PLOT Al_2_O_3_ S (50 m × 530 μm × 15 μm) capillary column. The X-ray diffractometer was from X’TRA, with CuKα irradiation. Analysis of light absorbance in solution was carried out on a Shimadzu UV-2700 UV-VIS Spectrophotometer.

**Figure 10:**
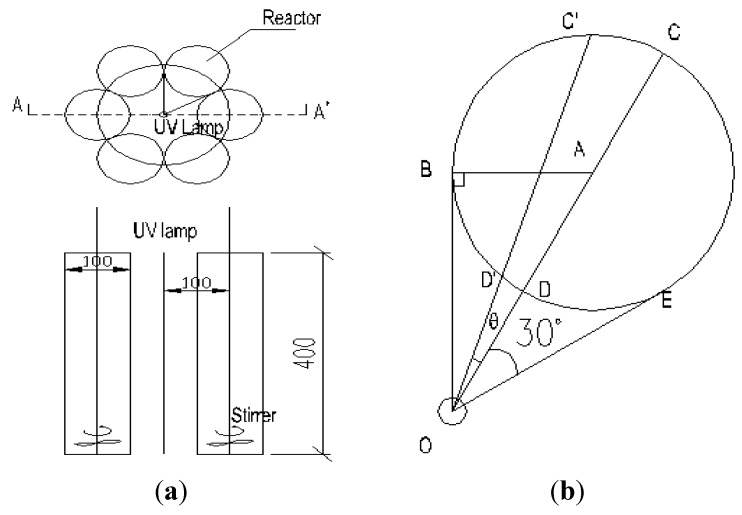
Demonstration of the photoreactor: **(a)** the Floorplan and A-A′cross sectional view of experimental reactor; **(b)** the outside irradiation model. C′D′ = L, OD = r_0_, O′D′ = r_1_, OA = 100 mm, AB = 50 mm, ∠AOE = 30°, θ = ∠AOC′

The exhibitive diagram of the photoreactor is shown in Figure 10. The experiments were performed using six glass cylindrical column reactors, the diameter and height of which were 100 mm and 400 mm, respectively. They were distributed symmetrically around the UV lamp(s). The working volume of each reactor was 3000 mL. The intensity per unit length, expressed as *S_L_*, was 400 W/m, except in Section 2.4. The lamps were located in the central of six cylindrical column photoreactors and externally irradiated the solution. The distance between the photoreactor surface and the lamps was 32 mm.

### 3.2. Pretreatment of Tourmaline Powder

The crude tourmaline powder was firstly passed through a 3000-mesh sieve. Then, the filtered powder was immersed in 1 mol/L HCl for 24 h. After that, it was washed repeatedly with double-distilled water till the supernatant turned neutral. Finally the powder was separated out and dried at 80 °C.

### 3.3. Synthesis of Tourmaline-Coated TiO_2_

Tourmaline-coated TiO_2_ was prepared using sol-gel method. The experiment was carried out in a room, the temperature of which was monitored and held constant at 25 °C using an air-conditioner. Firstly, 9 mL tetrabutyl titanate (TBOT) was mixed with 80 mL ethanol, and 1% in weight, of treated tourmaline was added in with further stirring of 30 min. After that, a mixture containing 10 mL ultrapure water, 5 mL HCl and 20 mL ethanol was dripped carefully into the system till the pH turned to 0.8. The stirring was kept for 24 h, and then 1 mol/L NH_3_•H_2_O was dripped in till the gel was formed. After being aged for 3 h, the gel was evaporated at 110 °C in a drying oven, and calcined at 450 °C in a muffle oven for 5 h. Pure TiO_2_ was prepared with the same method without the addition of tourmaline.

### 3.4. Photocatalytic Degradation of 2,4-D by Tourmaline-Coated TiO_2_

3000 mL 2,4-D solution of a certain concentration was transferred into the glass reactor. The reaction temperature was monitored and held constant at 25 °C using an air bath. A certain amount of complex photocatalyst was then introduced in. Immediately after the mixing of photocatalyst and solution, the lamp(s) was turned on, which was calculated as the start moment of reaction. All experimental processes were accompanied by vigorous stirring and air-pumping into the system. Samples (10 mL) were taken at certain time intervals using an injector, then centrifuged (at 16,000g for 10 min), and the supernatants were separated out and stored at −20 °C until analysis. The comparative studies on adsorption and photodegradation of 2,4-D, were carried out along the same procedure without UV irradiation, or without addition of photocatalyst. All experiments were performed in triplicate.

### 3.5. Analysis and Characterization of Photocatalyst

The carrier gas used in the GC analysis was helium, with a flow rate of 1.5 mL/min. The injection temperature was 250 °C. The column temperature was 50 °C firstly, held for 0.75 min. Then it rose to 120 °C at 20 °C/min, and further to 230 °C at 2 °C/min. At last the temperature rose to 290 °C at 10 °C/min, and kept for 10 min.

The XRD characterization of tourmaline and TiO_2_ complex photocatalysts were under the following conditions: The tube voltage and current were 40 kV and 40 mA, respectively. The 2-θ was measured from 10 to 70 degrees with a step size of 0.02 degree and scan rate of 10 s/step.

## 4. Conclusions

The novel photocatalytic material was prepared by sol-gel method, using tourmaline powder as the carrier of TiO_2_ nanoparticles. This photocatalyst showed significantly higher catalytic activity than the pure TiO_2._ Based on the research of the affecting factors, *i.e.*, the concentration of photocatalyst, the light intensity and the initial concentration of 2,4-D, a model for the photocatalytic degradation of 2,4-D by this complex photocatalyst was established. Results calculated from this model were close to those obtained from actual tests. This model might be useful in the design of such photochemical treatment processes using tourmaline coated TiO_2_ as a novel developed photocatalyst.
